# IR-GPT: AI Foundation Models to Optimize Interventional Radiology

**DOI:** 10.1007/s00270-024-03945-0

**Published:** 2025-03-26

**Authors:** Jacqueline L. Brenner, James T. Anibal, Lindsey A. Hazen, Miranda J. Song, Hannah B. Huth, Daguang Xu, Sheng Xu, Bradford J. Wood

**Affiliations:** 1 Center for Interventional Oncology, NIH Clinical Center, National Institutes of Health (NIH), Bethesda, USA; 2 Computational Health Informatics Lab, Institute of Biomedical Engineering, Department of Engineering Science, University of Oxford, Oxford, UK; 3 NVIDIA, Inc, Santa Clara, USA

**Keywords:** Artificial Intelligence, Foundation models, Mulitmodal data, Procedural optimization

## Abstract

Foundation artificial intelligence (AI) models are capable of complex tasks that involve text, medical images, and many other types of data, but have not yet been customized for procedural medicine. This report reviews prior work in deep learning related to interventional radiology (IR), identifying barriers to generalization and deployment at scale. Moreover, this report outlines the potential design of an “IR-GPT” foundation model to provide a unified platform for AI in IR, including data collection, annotation, and training methods—while also contextualizing challenges and highlighting potential downstream applications.

## Introduction

**Fig. 1:**
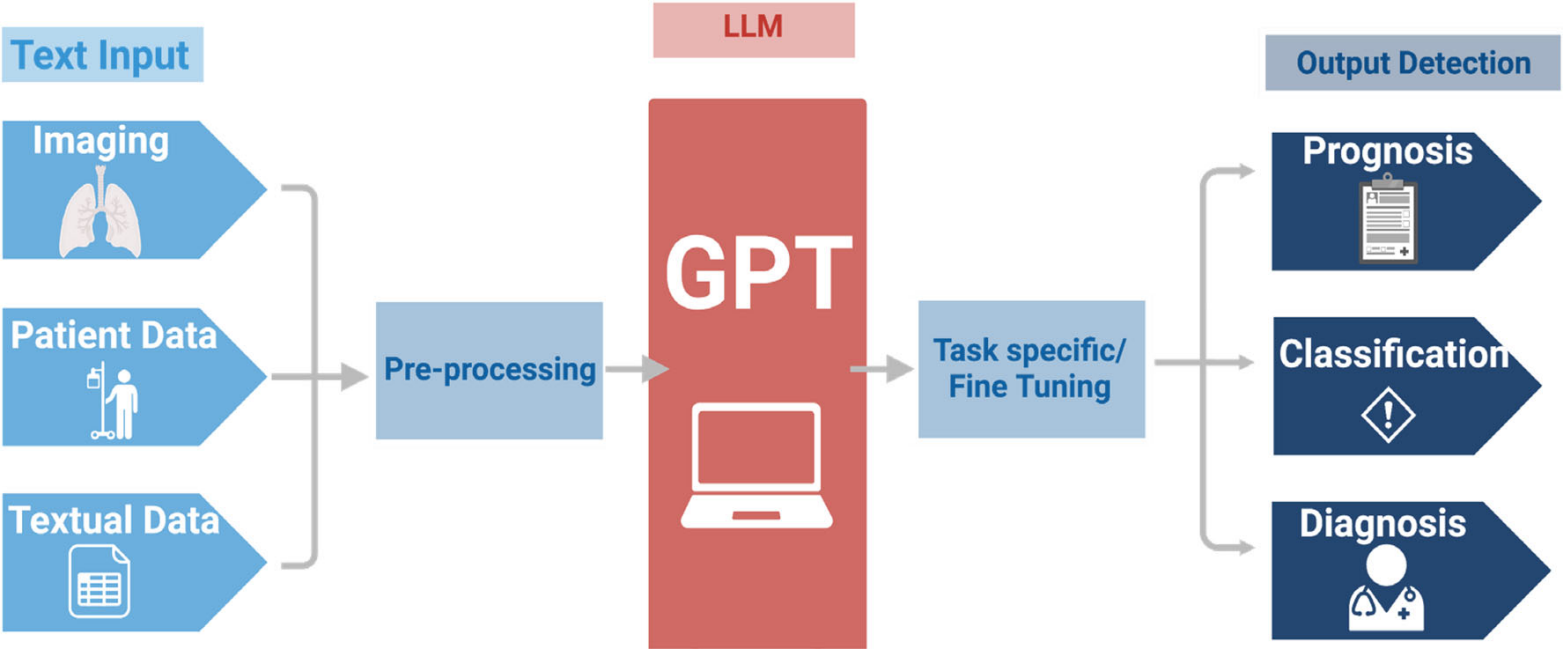
Sample workflow for a medical foundation model

Foundation AI models can learn complex information through self-supervised training on a vast amount of unlabeled data (code, text, images, etc.). With this advanced knowledge, foundation models like the generative pre-trained transformer (GPT) are able to perform diverse tasks on multimodal data inputs—including various spoken languages, coding languages, and mathematical expressions like differential equations [1, 2]. In the medical domain, GPT-3.5 recently outperformed physicians in both quality and empathy of responses to questions posted in an online health forum [3]. Large foundation models have also been built for computer vision, and have demonstrated capabilities in tasks involving medical imaging data (diagnostic radiology—DR, dermatology, pathology) [4–7]. However, these systems have not been specifically designed for applications related to image-guided minimally invasive procedures performed by interventional radiologists (Fig. 1). Yet, interventional radiology (IR) is highly dependent upon iterative imaging, with real-time human decision-making based on multimodal data [8]. These processes may be optimized with access to customized foundation models for real-time support of pre-, intra-, and post- procedural tasks [8–11].

### Foundation Models in IR

Conventional AI models often lack the essential medical domain knowledge and contextual understanding that is inherent to physicians; this is particularly true in complex procedural settings like IR [9, 12]. Expressive, domain-specific foundation models capable of processing the deluge of multimodal data may be a solution to the disorganized, insufficient AI ecosystem in IR, allowing physicians to ask questions, verify interpretations, and easily apply insights. Foundation models might address many unmet challenges in IR and may be able to inform decision-making in areas like treatment recommendations, targeted drug delivery, and device manipulations.

The initial step toward the deployment of foundation models in IR involves the identification of specific points in IR practice that represent safe, feasible opportunities for impact. This report aims to outline the potential structure and design of an IR-GPT framework, contextualize the challenges, and highlight potential applications of foundation models in IR.

## Past Work

Early efforts to apply AI in IR have been centered around the convolutional neural network (CNN), a deep learning model that uses the convolution operation to capture relationships between neighboring points in a matrix [13–15]. This algorithm has been frequently trained to perform tasks such as segmentation or binary classification of images from a single modality with limited datasets. Nevertheless, unique methodologies have emerged—examples include the incorporation of a 3D probabilistic deep learning system with CADe and CADx modules for lung cancer detection and the use of deep learning for prostate cancer detection through the development of a 3D U-Net and AH-Net trained on multiparametric MRI data [16–18]. CNNs in liver imaging or the two-stage cascaded CNN for Gleason scoring in prostate cancer were other innovations, concentrating on the discernment and categorization of areas of interest [19–21].

However, despite the apparent value of such methods for IR tasks, the current paradigm for AI in IR faces limitations. In many of the examples above, the model was trained to complete a single task, on a single organ system, using a single modality within a small dataset lacking diversity. To successfully build software that could support clinicians in standard IR procedures, a wide variety of scenarios and demographics must be considered, likely resulting in the need for hundreds—if not thousands—of conventional AI models to compose a generalizable system. This hypothetical system is not only unrealistic from a computational perspective but highlights the additional challenge of interpretability. The clinician would need to understand the outputs from many different models while also completing a procedure, creating additional technical burden. Existing work on foundation models in IR is quite minimal, focusing on tasks like generating simple reports, patient consent, and patient education (rather than procedural optimization) [22–25]. AI deployment in IR thus remains piecemeal. General categories of AI research in IR can be found in Table 1.

**Table 1:** Summary of Past AI research in interventional radiology

Research Area	Description
Robotics and Augmented Reality in IR	AI has be integrated into robotic and augmented reality systems to enhance the accuracy and efficiency of procedures involving these new methodologies
Vascular Imaging and Analysis	AI applications have been developed for analysis of vascular images—to predict deep venous thrombosis, massive pulmonary embolus, or risk for rupture of aneurysm. These tasks are crucial for planning and executing IR procedures or activating teams
Lesion Detection and Segmentation	AI algorithms have been developed to detect and segment lesions in imaging data used within the context of minimally invasive IR procedures, improving diagnostic accuracy and procedural planning. AI models have also been developed to automatically segment organs of interest, or needle pathways, such as transpedicular vertebral access
Predictive Modeling in IR	AI models have been used to predict factors like patient outcomes after IR procedures, tumors at risk for undertreatment, or personalized treatment volumes that may differ from a manual observer plan
Training and Education in IR Using AI	AI models have been used to enhance education related to IR, providing simulated environments for trainees and question-answering support for patients

In contrast with past examples, IR-GPT may be more multimodal and interactive, supporting healthcare professionals in completing a range of IR-specific tasks using different combinations of data inputs. IR-GPT may provide a singular channel for AI-driven enhancement of IR practice, compared to an overwhelming number of specialized smaller AI models that rely on unimodal inputs and focus on specific tasks like segmentation. Additionally, unlike conventional models, studies have shown that foundation models may be capable of few-shot or zero-shot learning—solving new, unseen problems different from those in training dataset [26]. This capability may facilitate robust performance on out-of-distribution inputs that may be commonplace in a rapidly changing healthcare environment like IR.

## Data Collection

Collecting adequate quantities of data is a major barrier to AI model development and applications in medicine, particularly in IR [27, 28]. Data requirements for a successful IR foundation model have created significant challenges in terms of annotation, cost, and privacy/ethics [27, 29–32].

### Data for Foundation Models

**Fig. 2:**
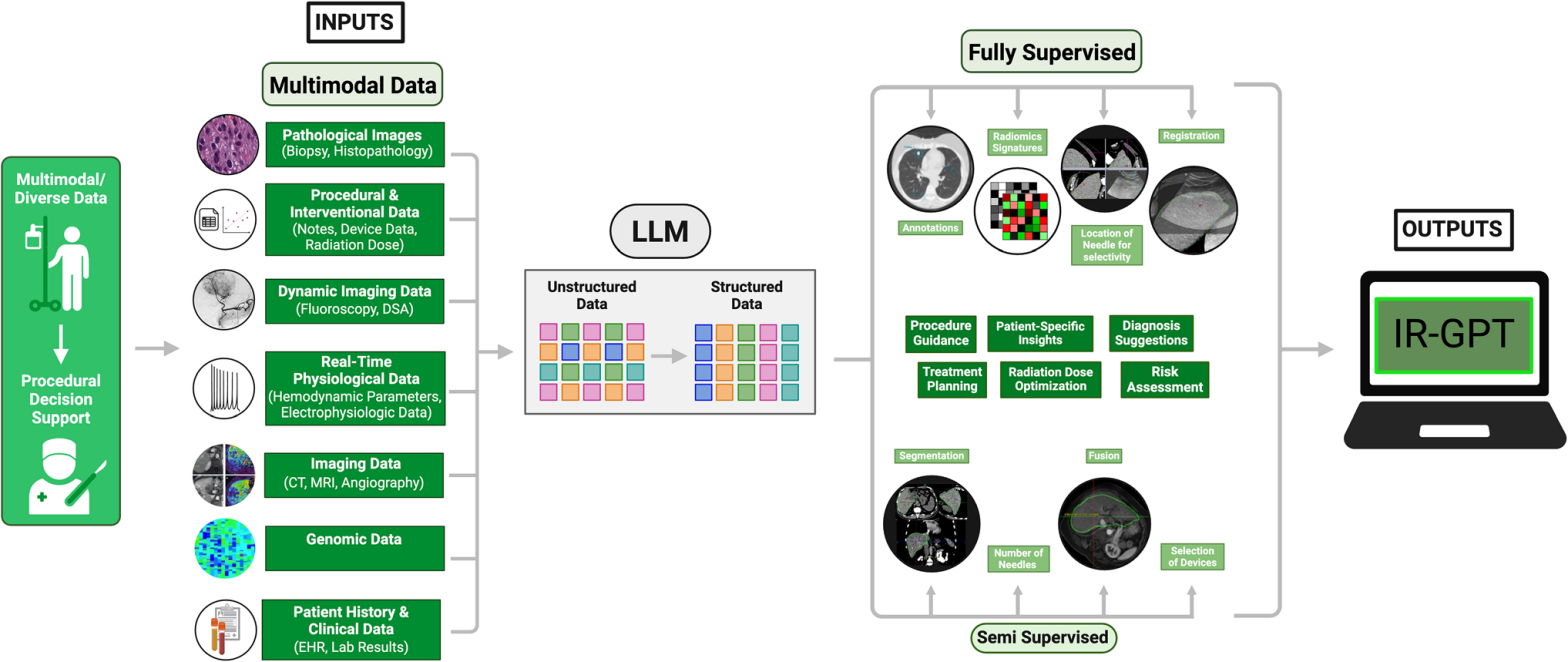
Training pipeline for the proposed IR-GPT method, including the input of multimodal data, the generation of prompts using audio data from procedures, and capabilities after training is complete

One major factor behind the recent success of foundation AI models, including large language models (LLMs), is the self-supervised pre-training on large datasets to encode general knowledge, which can then be fine-tuned for specific tasks. This is intuitive: the basic principles of the English language must be learned before passing radiology board exams [33]. In medical contexts, there have been numerous attempts to collect immense datasets which could be used to pre-train foundation models [13, 32]. Examples include the eICU database, MIMIC/MIMIC-CXR, NC3 COVID database, and MURA [34–38]. These datasets were mainly intended to train AI models for use in critical care and diagnostic radiology. In IR, procedures are substantially data-driven; yet, there have been few attempts to collect multimodal databases of longitudinal data that might be suitable for building foundation models related to procedural tasks [29]. This report proposes the development of an image-text-audio dataset containing IR-specific data that would be structured to ensure that AI algorithms could understand procedures across time and space. Such a dataset may possibly result in a customized foundation model for optimizing image-guided minimally invasive procedures: “IR-GPT” (Fig. 2).

### Large Datasets in IR

There are significant obstacles to assembling IR datasets for customized foundation models like IR-GPT. First, a substantial number of cases is required to establish a dataset sufficient for training an IR-GPT model. However, IR represents a small fraction of cases relative to all medical specialties. Establishing a vast repository of cases would require cooperation and data sharing between different healthcare systems, which is difficult due institutional/corporate competition and legal restrictions on data sharing [28, 29, 39, 40]. Further, uniformity is lacking in the planning, performance, and reporting of IR procedures [41].

There are other structural challenges associated with building large IR datasets. Data diversity is lacking in many current clinical datasets—these are typically skewed toward patients from relatively high-income areas [29]. This results in models which generate outputs that are biased and even potentially dangerous for under-represented groups [30]. IR datasets include live procedural data and contextual information such as electronic health records (EHR), introducing possible biases including race, gender, device quality, and socioeconomic status. Diverse datasets are needed to reduce the risk of these harmful biases [42]. Foundation models trained on diverse data will have an enhanced understanding of patient-specific conditions, perhaps facilitating more equitable and precise IR practice. However, to accomplish this goal, data must be collected from healthcare facilities in diverse settings with varying technological ecosystems.

Compared to other medical specialties, collecting complex data from IR procedures presents additional challenges. Many healthcare systems lack the funding and infrastructure needed to continuously collect and annotate multimodal data from live procedures [30, 42]. For example, procedural images would require corresponding descriptive text or other labels—without this key information, the model would have no basis for calculating error and improving performance. Unlike most diagnostic radiology models, AI systems for IR must be evaluated at multiple time-points throughout a procedure.

### Pathways to IR-GPT: Data Collection

A centrally coordinated and funded consortium is one potential solution to the challenge of building a dataset for IR-GPT. For example, the NIH “All of Us” program seeks to establish a balanced and representative dataset for medical research in genomics through multi-institute partnerships [43].

A similar program for AI in IR, particularly focusing on the development of foundation models, could overcome several of the above challenges by supporting infrastructure for a data collection and sharing across diverse healthcare environments. This could help ensure that resource-constrained healthcare facilities have the funding to contribute data from underserved communities. Cross-disciplinary teams of AI researchers, data scientists, and IR clinician scientists could pursue multi-center efforts to build the tools for an IR-GPT model. Additionally, initial efforts have been started to collect crowdsourced data and questions (with annotations/answers) for training IR-GPT. Crowdsourcing would ensure that IR-GPT is not limited by the specific, potentially narrow perspectives of a single group/institution—there are often multiple approaches to a procedure, and not all situations are covered by standardized guidelines.

#### Data Annotations

Rather than relying exclusively on image-text pairs, data for IR-GPT may be annotated via semi-structured “procedural narrations” in which a healthcare worker describes (with speech) the key steps of the procedure, providing explanations for decisions/actions. Guidelines may be used to ensure some level of standardization between annotators. This work could be done in real-time or retrospectively by IR annotation teams that could simulate procedures using existing multimodal data.

Routine parts of the procedure may be annotated as neutral or a continuation of a past state based on a lack of audio, reducing the time and burden required to annotate training data for IR-GPT. The care team or annotator would likely need to record only several minutes of narration  per procedure. Recorded audio data could also be collected for pre-procedure treatment decisions, such as “tumor board,” integrating information from history, past therapies, pathology, imaging, EHR, and labs. Data from recorded videos of the operating room (OR) have been proposed previously for the development of AI models involving procedural data, but audio data are less invasive, simple to transcribe, affordable to store at scale, and easily anonymized to protect privacy [44]. These narratives could also be used by healthcare professionals in the process of post-procedural notetaking or reviews/debriefings to improve skills/teach trainees.

To use procedural narration data for training IR-GPT, expert clinicians may first curate lists of queries that would frequently be relevant within an IR workflow. At each key step in the procedural process, an LLM would be used for converting narrative information into a target response that the model would aim to replicate. By providing supervised material for preliminary training, this annotation method ensures that the model has an IR-specific knowledge base, which may simplify subsequent model refinement involving unlabeled retrospective data and human AI trainers.

## Model Training

Current applications of AI in IR may require the use of many separate models to support a single procedure, including tools for tasks such as triage, sequential organ segmentation, tumor segmentation, treatment volume segmentation, and registration for targeting, monitoring, verification, and follow-up. This section proposes strategies for training an IR-GPT model to unify the ecosystem.

### IR-GPT

Foundation models that are customized for IR applications—“IR-GPT”—may address current challenges by providing a unified system that learns from complex multimodal data (regardless of input structure), completes tasks from instructional prompts, and provides clear outputs for expert consideration. Transformer-based models complete tasks based on an aggregated representation of relationships between combinations of features, and are therefore invariant to the size, order, or composition of the input [45]. IR-GPT, through effective integration of different data modalities (e.g., imaging, text, EHR, audio) may facilitate the practical deployment of AI tools in interventional radiology settings, including procedural or other image-guided environments. Existing large language models (LLMs) have already demonstrated potential in some limited applications, including personalised patient education prior to interventional radiology procedures [22]. IR-GPT may enable more advanced capabilities.

### Training Methods for IR-GPT

The training of IR-GPT will be based on a large corpus of multimodal and multisite data labeled with “procedural narrative” audio recordings provided by an interventional radiology care team or annotation team (as described in Sect. "Data Annotations"). This will directly involve the use of a pre-existing LLM to generate instructional prompts (questions) and outcomes based on reports and recorded narrative data (audio with time-paired images).

#### Pre-Training

The initial weights of IR-GPT could be obtained from existing multimodal foundation models that were trained on large (unlabeled) image, text, and clinical datasets. The basic knowledge of medicine learned from this initial phase will improve the subsequent IR-specific components of model training by capturing general patterns, features, and relationships that may be generally relevant across different subspecialties.

#### Supervised Learning

For each timepoint in the procedural sequence (including pre-, intra, and post-procedure settings), data in the form of questions (with patient-specific context) could be input into the model. The IR-GPT model would then output a response, which would be automatically compared to the procedural narration data structured by the LLM (Fig. 2). For example, an ideal response to “given the following contextual information *[case],* what device, technique, approach, imaging modality, and perspective would be best?” could be, “a cobra catheter and a coaxial micro-catheter system, with an angled glide wire and a 45-degree micro-catheter at 48 degrees LAO with 8 degrees cranial tilt to the detector and fusion guidance with 3D fluoroscopy referencing PET data.” Optimization functions could be used to adjust the model weights based on a cost function (evaluating similarity to the desired answer).

#### Reinforcement Learning with Human Feedback

After supervised training, which captures initial IR-specific knowledge, large quantities of unlabeled retrospective data from past procedures could be leveraged to refine the model via insight from IR professionals. This might expand the breadth and depth of information encoded within IR-GPT. Here, similar to the training protocol for existing LLMs, reinforcement learning from human feedback (RLHF) may be used to optimize the system based on insights from human trainers who rank the outputs of the model at each step [46]. Past work has demonstrated the effectiveness of RLHF in improving model performance, and the ranking process is also more efficient than complete audio annotation of procedural data [46]. The team of human trainers for the IR-GPT model may include a distributed network of healthcare workers (experts), medical students, and researchers.

## Deployment of AI in IR

Many decision points in IR procedures might be better informed with support from customized foundation models. Areas of impact might include decisions on catheter–wire combinations and complex detector angles to match known anatomy or vascular branching points. Detection of complications like pneumothorax, bleeding, or endoleaks might be quantified by an IR-GPT model that was fine-tuned for risk assessment. Optimal needle pathways might be predicted by the model with aim of avoiding pleural fissures or arterial anatomy. Endpoint detection for TACE/TARE or ablations might be informed by automated virtual perfusion and margin detection; this could also be enabled by an LLM that can extract information from imaging data. Moreover, with multimodal capabilities, a consolidated assessment of cardiac function and EHR historical parameters might enable the auto-detection of an acute pulmonary embolus, triggering the activation of an emergency team. Other processes like tumor boards might be more cost-effective when guided by a foundation AI model with access to fact-based medical records, high-level evidence guidelines, and imaging data. Finally, IR professionals will undoubtedly discover new uses which add value in clinical settings, through different prompting strategies, data inputs, or wrapper applications.

**Fig. 3:**
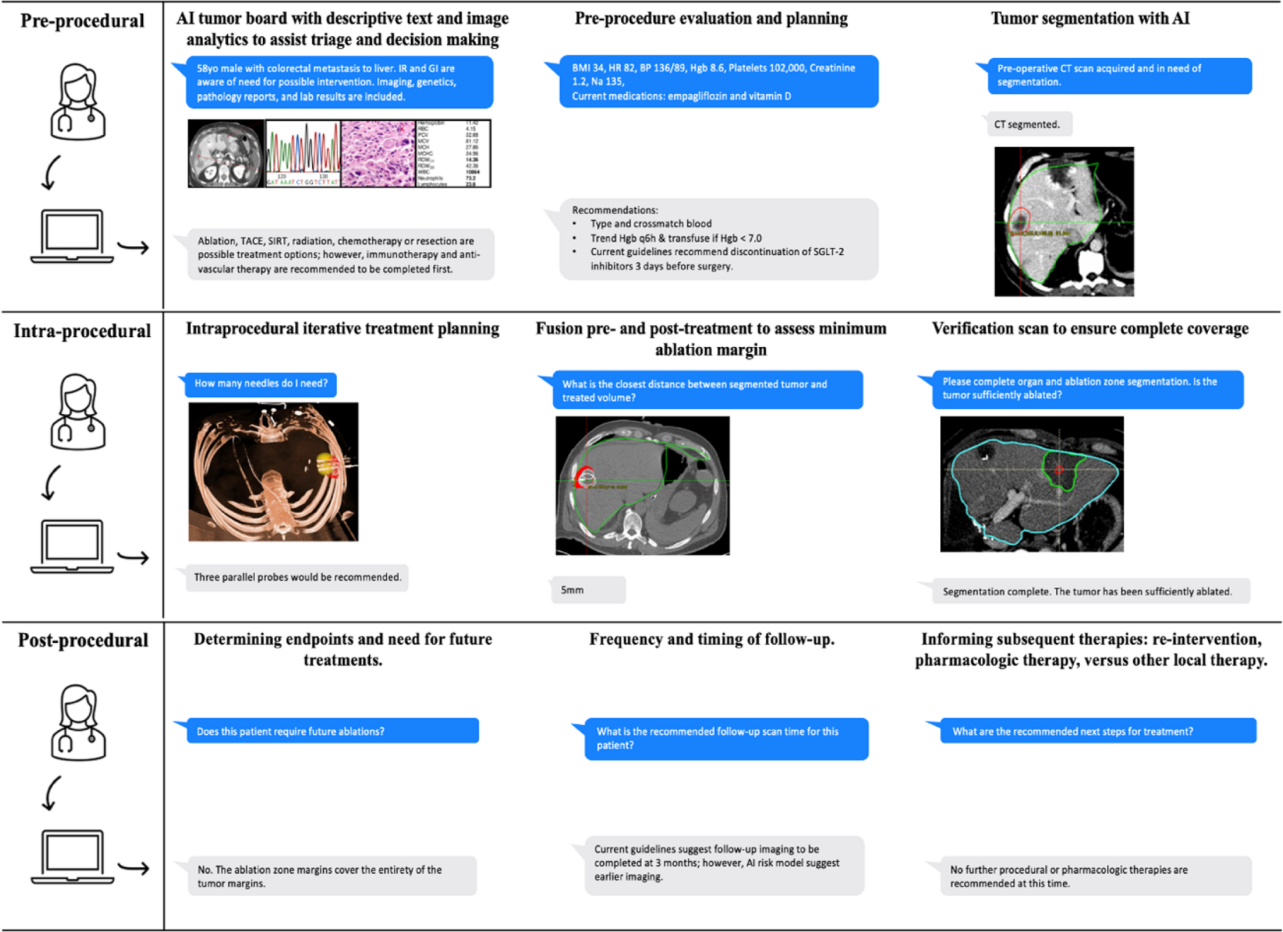
Pre-procedural, intra-procedural, and post-procedural use cases of IR-GPT

In Fig. 3, various uses cases of IR-GPT are illustrated, including pre-procedural, intra-procedural, and post-procedural applications (Fig. 3). During the procedure, a trained IR-GPT model may quickly and clearly inform clinicians about selection and use of surgical tools, delivery of therapeutics, and outcomes of procedural components—thereby enhancing patient outcomes.

## Clinical Challenges and Limitations

Past work has shown that AI models may have vulnerabilities and biases from the training data. To ensure that models can provide consistent performance across settings with variable resources, including in low- and middle-income countries (LMICs), data and audio annotations must be collected from diverse environments. IR-GPT must also be validated with data in different languages, particularly if machine translation is used to ensure compatibility with the model. Another key challenge may be the ethical and privacy concerns that must be considered during the curation of large IR training datasets. Anonymization techniques may be required to ensure all data are non-identifiable, inadvertent protected conversations, and other sensitive information from the procedural narratives.

Even after the development of IR-GPT, there may be limitations in the outputs that could pose challenges to use in real-world clinical settings. For example, responses similar to those in Fig. 3 may be insufficiently detailed or require multi-turn interactions to obtain the necessary information. Lengthy engagements with an AI model would likely create delays that are highly inpractical for an IR suite. This constraint is unique to fast-paced clinical settings—chatbot models for basic dialog often rely on multi-turn conversations to provide accurate responses. Finally, despite crowdsourcing and multi-site efforts, an IR-GPT model may still have biases against techniques or products which are not used by a majority of IR healthcare workers (e.g., due to rare cases, resource limitations, or variabilities in devices/vendors).

## Conclusion

The future implementation of an IR-specific foundation model has significant potential to impact clinical practice, particularly in settings with IR education and expertise [47]. This report proposes a more comprehensive pathway to develop AI solutions that address existing limitations in this dynamic and technology-oriented space. Undoubtedly, these advanced AI technologies for data-driven decision support—very possibly in the form of an IR-GPT model—will shape the future of IR.
